# The role of immunosuppression of mesenchymal stem cells in tissue repair and tumor growth

**DOI:** 10.1186/2045-3701-2-8

**Published:** 2012-03-05

**Authors:** Zhipeng Han, Yingying Jing, Shanshan Zhang, Yan Liu, Yufang Shi, Lixin Wei

**Affiliations:** 1 Tumor Immunology and Gene Therapy Center, Eastern Hepatobiliary Surgery Hospital, the Second Military Medicial University, Shanghai, China; 2 Institute of Health Sciences and Shanghai Institute of Immunology, Chinese Academy of Sciences and Shanghai Jiao Tong University School of Medicine, 225 South Chongqing Road, Shanghai, China

**Keywords:** Mesenchymal stem cells, Immunosuppression, Tumor growth

## Abstract

Mesenchymal stem cells (MSCs) have acquired great interests for their potential use in the clinical therapy of many diseases because of their functions including multiple lineage differentiation, low immunogenicity and immunosuppression. Many studies suggest that MSCs are strongly immunosuppressive *in vitro *and *in vivo*. MSCs exert a profound inhibitory effect on the proliferation of T cells, B cells, dendritic cells and natural killer cells. In addition, several soluble factors have been reported to involved in the immunosuppressive effects by MSCs such as TGF-β, HGF, PGE2, IDO and iNOS. These results suggest that MSCs can be used in the therapy of immune disorder diseases, prevention of organ transplantation rejection and tissue injury. In recent study, we demonstrated that MSCs in tumor inflammatory microenvironment might be elicited of immunosuppressive function. Thus, the application of MSCs in cancer therapy might have negative effect by helping tumor cells escaping from the immune surveillance.

## Introduction

Bone marrow is the most important source of adult stem cells, which contains heterogeneous populations of cells including hematopoietic stem cells, erythrocytes, fibroblasts, adipocytes and etc. Mesenchymal stem cells (MSCs) is a subset of nonhematopoietic stem cells existed in bone marrow originating from the mesodermal germ layer[1,2]. MSCs were first identified by Friedenstein and were described as an adherent, fibroblast-like population in the in vitro culture of bone marrow which were also found to be able to differentiate into bone in vivo[3]. MSCs also existed in other tissues, including adipose, umbilical cord, fetal liver, muscle and lung [4-8].

MSCs have the ability to differentiate into multiple lineages such as chondrocytes, osteocytes, adipocytes, myocytes, and astrocytes, so MSCs could be considered as a potential source of stem cells for cellular and genetic therapy [5,9]. The phenotype of MSCs is identified by the absence of the CD34 and CD45 hematopoietic cell markers and is positive for CD29, CD90 and CD105[10]. MSCs express the major histocompatibility complex (MHC) class I but do not express MHC class II, B7-1, B7-2, CD40 and CD40L molecules. MSCs can be expanded more than 10^4^-fold in culture without loss of their multilineage differentiation potential. Some studies have established that bone-marrow-derived MSCs can engraft injured tissues, such as those of the lung, liver, heart, and brain, and recover their function. In past ten years, many studies have confirmed that the use of stem cell transplantation is an important tool in the treatment of several types of malignancies[11-13]. For these reasons[14], such cells are currently being tested for potential use in cell and gene therapy for tumors[15,16]. In contrast, newer studies have proposed that stem cells may be the direct cellular targets of the genetic alterations that lead to tumor formation and important contributors to the maintenance of human cancers[17,18]. Emerging evidence suggests that both bone-marrow-derived MSCs and mature stromal cells can play an important role in the growth and development of human malignancies[19,20].

### The immunosuppressive properties of MSCs

In recent years, more and more researchers pay attention to the immune modulatory property of mesenchymal stem cells and a large amount of researches have shown that one of the most important properties of MSCs is the immunosuppressive properties. As immunomodulators, MSCs may play important roles in many diseases with immune disorder[21]. Accordingly, it is very important to explore the molecular mechanisms of immunosuppressive properties.

### Mesenchymal stem cells and T cells

Several studies have demonstrated that MSCs can exert immunosuppressive effect on T cells, which are a major executor of the adaptive immune response. T cells anergy induced by MSCs has been regarded as a potential mechanism of immune suppression. The proliferation of T cells induced by mitogens, alloantigens and CD3 and CD28 antibodies is significantly inhibited by MSCs [22-25].

MSCs lack surface expression of costimulatory molecules, such as CD80 (B7-1) and CD86 (B7-2), and constitutively express MHC-I while the expression of MHC-II is relatively low or absent [22,26,27]. The inhibition from MSCs affects several aspects associated with the function of T cells including activation, antigen-specific proliferation, cytotoxic T cells (CTLs) formation, down-regulation of IFNγ in Th1 cells and up-regulation of IL-4 in Th2 cells[24,28]. Potian et al [26] demonstrated that MSCs could inhibit the cytotoxic effects of antigen-primed CTLs and the internal mechanism is due to suppression of the proliferation of CTLs, rather than an inhibition of cytolytic activity[29,30].

It has been believed that soluble factors contribute to MSCs immunesuppression because MSCs demonstrate inhibition of PBMCs proliferation in a transwell system [22,25,30]. However, conditioned culture medium collected from MSCs does not illustrate inhibitory effect unless they are cultured with lymphocytes indicating that the production of immunesuppressive factors in MSCs requires a cross-talk between MSCs and lymphocyte[26,31,32].

Several soluble factors are involved in MSCs-mediated immunomodulation including transform growth factor-β (TGF-β), hepatocyte growth factor (HGF), prostaglandin E2 (PGE2), soluble HLA-G5, heme oxygenase (HO)-1, indoleamine 2, 3-dioxygenase (IDO) and inducible nitric-oxide synthase (iNOS)[33-36]. The soluble factors are produced by MSCs constitutively or released after MSCs cross-talk with target cells. It is important to point out that the factors responsible for suppressing T cells vary depending on the experimental system and in vivo situations.

Furthermore, some studies describe the toll-like receptors (TLRs), which are conserved family receptors that recognize pathogen-associated molecular patterns and promote the activation of immune cells, are involved in the immune regulatory effects MSCs[37,38]. Pevsner-Fischer et al. [38] demonstrated that expression of TLRs molecules 1 to 8 is positive in MSCs and TLRs ligands could effectively activate MSCs to produce IL-6 and lead to NFκB nuclear translocation. Nemeth et al. [39] reported that MSCs activated by LPS or TNF-α could reprogram macrophages by releasing PGE2. These results suggest that TLRs and TNFR expression in MSCs is associated with the immunosuppression.

### Mesenchymal stem cells and B cells

Murine MSCs have been reported to have the inhibitory effects on B cells in terms of proliferation, activation and IgG secretion[40]. Furthermore, human MSCs could also inhibit B cell proliferation. In addition, MSCs lead to the downregulation of chemokine receptors CXCR4, CXCR5 and CCR7 in B cells, which indicated that MSCs could affect the chemotactic properties of B cells [41]. The phenomenon that soluble factors released by MSCs were sufficient to inhibit proliferation of B cells in Transwell experiment while the culture supernatant from MSCs had no effect, suggested that the production of inhibitory soluble factors in MSCs needs paracrine signals from B cells.

### Mesenchymal stem cells and dendritic cells

Dendritic cells (DCs) play a key role in the initiation of primary immune responses and in tolerance, depending on the activation and maturation stage of DCs[42]. On one hand, they contribute to activating naive T-cell during the primary immune response. On the other hand, they are also involved in activation of B cells by soluble factors or Th cells. So DCs are extremely important not only in cellular immunity but also in humoral immunity[43].

Emerging evidence suggested that MSCs could inhibit the activation and maturation of DCs. MSCs have been reported to reduce the activation of T cells by reducing the formation of DCs from monocytes [29]. MSCs could inhibit the differentiation and function of DCs, as indicated by transwell assay. Furthermore, the DCs which were co-cultured with MSCs lost the potential to activate CD4+ T-cells in MLC[44]. MSCs can down-regulate the expression of CD40, CD1a, CD80, CD86 and HLA-DR during the differentiation of monocytes to DCs and suppress CD83 expression which associate with DCs maturation [28,29,45,46].

MSCs are reported to reduce the production of several cytokines in DCs, such as IL-12 and TNF-α. MSCs decrease the function of DC to secrete IL-12, which is important to promote an effective cellular immunity by activating and differentiating T cells[45]. The decrease of TNF-α secretion DC1 and increase of IL-10 in DC2 caused by MSCs may lead to a state of immune tolerance. IL-10, characterised by immunosuppression, can significantly influence DCs function in many aspects. IL-10-producing DCs lose their function to stimulate lymphocyte while effectively inhibited the proliferation of T-cells[47-49]. PGE2 has also been observed to involve in the modulation of DCs maturation. Blocking the production of PGE2 in MSCs could delete their inhibitory influence on DCs [50]. Djouad et al demonstrate that high levels of IL-6 expressed by MSCs can lead to a transformation of mature DCs to a less mature phenotype[51].

### Mesenchymal stem cells and NK cells

Natural killer (NK) cells are major effector cells of the innate immunity and play a key role in antiviral responses[52]. NK cells mainly exhibit cytolytic activity on the cells that lack expression of HLA class I molecules. The killing function of NK cells is controlled by the activating and inhibitory receptors interacting with HLA molecules on target cells. The autologous cancer cells are probably lysed by NK cells [53]. Several studies have reported the interaction between MSCs and NK cells [54-56]. It has been demonstrated that MSCs could effectively inhibit IL-2-induced proliferation of resting NK cells, whereas the proliferation of activated NK cells is partially affected. Furthermore, IL-2-activated NK cells (but not freshly isolated NK cells) efficiently lyse autologous and allogeneic MSCs [55]. The major receptors of activating NK cells, NKp30, NKG2D and DNAM-1 play a key role in NK cell-mediated cytotoxicity against MSCs. The ligands of these activating NK receptors, ULBPs, PVR and Nectin-2, are also expressed by MSCs.

Soluble factors such as TGF-β1, PGE2, and IDO may play an important role in MSCs-mediated inhibition of NK cells function [22,28,56,57]. The mechanisms underlying the immunosuppressive effects of MSCS are still unclear. On one hand, Sotiropoulou et al [56] show that PGE2 secreted by MSCs can partially affect NK cells proliferation, CD56 expression and cytotoxicity without interfering cytokine production or expression of activating receptors of NK cells. The proliferation of NK cells is partially restored by inhibition of TGF-β1, while combined blocking PGE2 and TGF-β1 will lead to a complete recovery of NK cells proliferation. On the other hand, Spaggiari et al [55] reported that MSCs could effectively inhibit the proliferation of NK cells and the simultaneous blocking of IDO and PGE2 could almost completely restore NK-cell proliferation. Furthermore, MSCs could markedly reduce killing ability of NK cells on target cells [54].

### Inflammatory microenvironment and immunosuppression of MSCs

In the past, series of studies on MSCs immune modulatory properties were carried out in animal and human with immune disorder disease[58,59]. Since the experiment result that allogeneic MSCs are not rejected when administered into baboon is achieved, the immune tolerance mechanism have been confirmed [60,61]. From lots of *in vivo *studies, we know that MSCs can effectively suppress immune response. But some contradictory phenotype can be observed, which is an effective immunosuppression can be achieved in some *in vitro *experiments, but *in vivo *experiment MSCs injection do not prolong allograft survival[62]. For instance, some studies showed that MSCs cannot prolong graft survival in vivo despite that they could suppress lymphocyte proliferation *in vitro*[63]. Some other studies also demonstrated the similar results that MSCs could not prevent graft rejection *in vivo*. To explain this phenomenon, we detected the effects of mouse MSCs on T cells proliferation. The results indicate that indicate that there is no difference in the proliferation of T cells blasts driven by IL-2 with or without MSCs coculture[64,65] neither the T cells hybridoma(A1.1). Therefore, we conclude that MSCs do not suppress T cells proliferation without the T cells activation. Based upon the above results, a question was raised why immunosuppressive function of MSCs is dependent on T cells activation.

To resolve this question, we culture MSCs with A1.1 cells or T cell blasts with the supplementation of supernatant from anti-CD3-activated T cell cultures. The result showed that T cell proliferation was significantly inhibited, which suggested that some products of activated T cells played important roles in priming the immunosuppression capacity of MSCs. Subsequent studies indicate that proinflammatory cytokines are needed to induce the immunosuppression function of MSCs such as IFN-γ, TNF-α, IL-1α or IL-1β. IFN-γ is essential and TNF-α is the product of T cell. IL-1α and IL-1β are the products of APCs [66]. Inflammatory cytokine activated MSCs can inhibit the proliferation of many types of immune cells, including freshly-isolated anti-CD3-activated splenocytes, purified CD4+ T cells, CD8+ T cells or plastic-bound anti-CD3 activated T cell blasts, anti-CD28, and LPS-activated purified B cells or macrophages. We also found that the activated MSCs can inhibit the proliferation very efficiently as the ratio one to 50 or 100 T cells. Therefore, the immune modulatory properties of MSCs need the priming by inflammatory cytokines. After making the above question clear, we were drawn to another question which we cannot explain. We found that T cells proliferation cannot be suppressed when MSCs and T cells are cultured by transwells or physical separation separately. Relevant researches showed that the immunosuppressive effect of mouse MSCs was completely diminished when inducible nitric oxide synthase (iNOS) was inhibited by selective inhibitors, NG-monomethyl-L-arginine (L-NMMA), N-[3-(Aminomethyl)benzyl] acetamidine, Dihydrochloride (1400 W) or NG-Nitro-L-arginine Methyl Ester, Hydrochloride (L-NAME). In addition, MSCs derived from mice deficient in iNOS did not inhibit T cells proliferation. Consequently, NO is an effector molecule in suppressing immune reactions by inflammatory cytokine -primed MSCs. Furthermore, NO can only affect the cells near to it because of the biological activity diminishes within a distance of a few cell diameter.

### The role of MSCs in tissue injury repair and immune disorder disease

Since they have immunosuppressive property, MSCs were investigated as a new potential therapy for some immune disorder diseases, such as graft-versus-host disease (GvHD)[67,68], Crohn's disease[69], and the prevention of organ transplantation rejection[70]. Furthermore, many studies have demonstrated MSCs play a critical role in injury healing [16,58,71-73]. MSCs transplantation is also regarded as a useful therapeutic strategy in acute tissue injuries of the lung[74], heart[75] and liver[76-78]. Although the lack of a reliable surface marker for MSCs hinder the identification of MSCs that migrate to injury site, we still think it is reasonable to suppose that MSCs may be mobilized and recruit to a severe wound site[79]. On one hand, MSCs may repair tissue injury by differentiation into other kinds of cells; on the other hand, as a part of microenvironment, MSCs support the growth of other cells, such as fibroblasts, endothelial and epithelial cells, macrophages, neutrophils and lymphocytes.

Our previous study has showed that IFN-γ and NO production were critical for immunosuppression of MSCs in GvHD model[66]. MSCs can inhibit T cell response and decrease inflammatory cytokines production in mouse rheumatoid arthritis (RA) model[80]. In experimental autoimmune encephalomyelitis (EAE), MSCs were also observed to have a therapeutic effect on infiltration of T cells in central nervous system (CNS)[23].

### Role of MSCs immunosuppressive function in tumor growth

**Figure 1:**
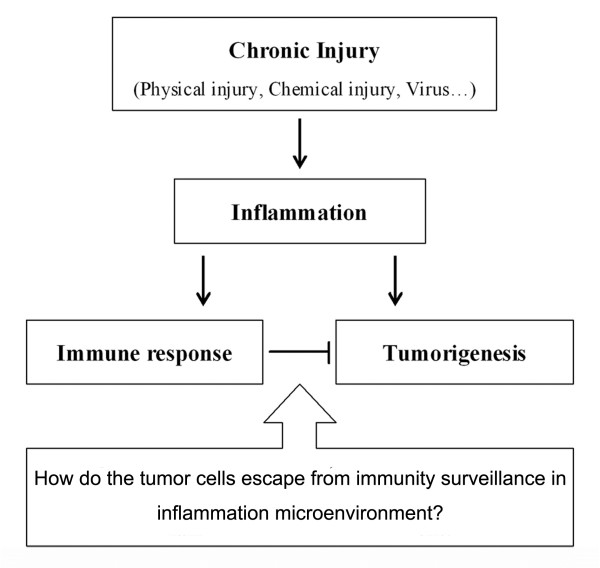
**Chronic inflammation associated with the whole process of tumor occurrence and development**. However, the presence of immune response in inflammation is concerned as a negative factor for tumor growth. The contradictory phenomenon brings us a problem how tumor cells escape from immune destruction in an inflammatory microenvironment?.

Chronic inflammation is associated with the whole process of tumor occurrence and development. However, immune response in the inflammatory environment of tumor could suppress tumor growth. The contradictory phenomenon brings us a problem which is how tumor cells escaping from immune destruction in an inflammatory microenvironment?(Figure 1)

**Figure 2:**
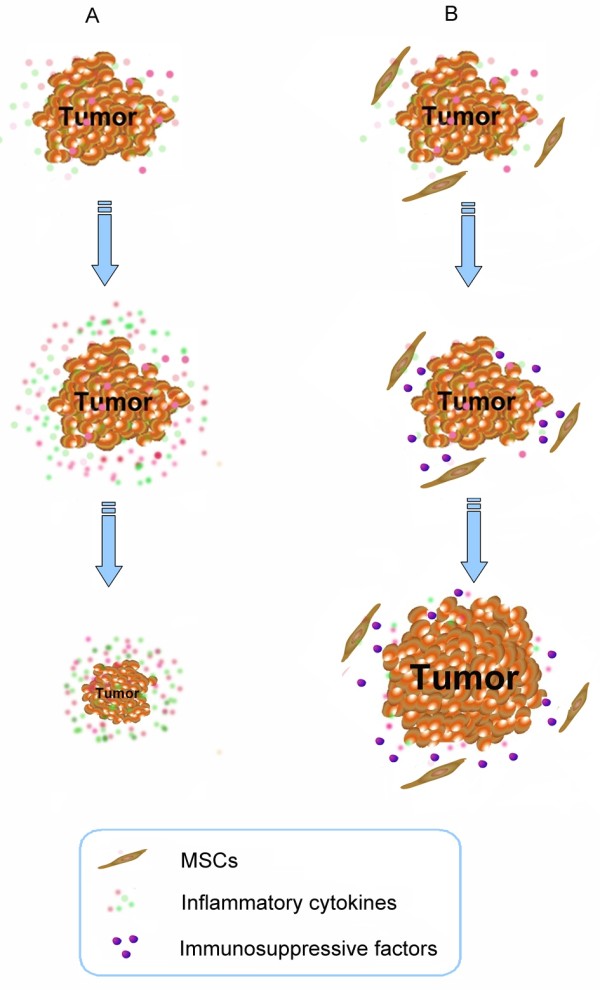
**MSCs have a tropism for tumors**. We have demonstrated that inflammatory cytokines could effectively induce immunosuppression of MSCs. Our results suggest that the MSCs in tumor inflammatory microenvironment may be elicited of immunosuppressive function, which will help tumor to escape from the immunity surveillance.

MSCs play an important role in treating various degenerative diseases and immune disorders. They have great potential for correcting aberrant immune reactions. Previous studies showed that these cells could be expanded and induced ex vivo, terminally differentiate into osteoblasts, chondrocytes, adipocytes, myotubes, neural cells and hematopoietic supporting stroma[5,9,81]. The immunosuppression demonstrated by MSCs has been reported in several studies [22,24,36,82]. However, in certain circumstances the property of immunosuppression may display negative effects such as the promotion of tumor growth. Ren et al. have shown that the immunosuppressive function of MSCs is elicited by proinflammatory cytokines and the immunosuppression of MSCs is through the concerted action of chemokines and NO[66]. MSCs have a tropism for tumors[83] and the incidence and development of tumor is always accompanied with proinflammatory cytokines. So it is important to investigate the effect of MSCs favoring tumor growth in inflammatory environment. To investigate this hypothesis, we assessed the role of MSCs in inflammatory environment on the development of tumor by B16 melanoma cells implanted in allogeneic mice. Our results suggest that the MSCs in tumor inflammatory microenvironment may be elicited of immunosuppressive function, which will help tumor to escape from the immunity surveillance[84](Figure 2).

Recent studies have shown that MSCs displayed an ability to favor tumor growth under certain circumstance. Djouad et al. [32] showed that MSCs had displayed side effects related to systemic immunosupression favoring tumor growth in vivo. Hall et al. [85] have also shown that co-culture of ALL cell lines with stromal cells which is overexpressed of VCAM-1 enhanced survival of leukemic cells in a PI-3 kinase dependent manner, compared to the co-culture with stromal cells expressing only endogenous VCAM-1. On the contrary, MSCs have been reported to be anti-tumorigenic in a mouse model of Kaposi's sarcoma by inhibiting AKT activity [20]. Our study implies that inflammatory cytokines may be the key factors that regulate the effect of MSCs on tumor growth. The presence of an active immune reaction in tumor environment can lead the MSCs that localized in tumor to acquire immunosuppressive function. On the other hand, the absence of active inflammation in tumor may not only abstract MSCs to localize but also induce them to be immunosuppressive.

## Conclusions

Because of a wide range of MSCs function including multiple lineage differentiation, low immunogenicity and immunosuppression, have acquired many interests for their potential use in therapy of many clinical diseases. We can expect an exciting result for the application of MSCs in some immune disorder diseases, prevention of organ transplantation rejection and tissue injury. It should be noted the study of MSCs clinical application needs to pay attention to the local microenvironment. Our results suggest that the MSCs in tumor inflammatory microenvironment may be elicited of immunosuppressive function. Although MSCs have a tropism for tumors and some studies reported to used MSCs as a drug carrier for tumor therapy, the immunosuppression of MSCs may be a risk to help tumor escaping from the immunity surveillance. Therefore, the use of MSCs in cancer therapy should be extremely cautious.

## List of abbreviations

MSCs: mesenchymal stem cells; MHC: major histocompatibility complex; CTLs: cytotoxic T cells; TNF-α: tumor necrosis factor-alpha; HGF: hepatocyte growth factor; IFN-γ: interferon-gamma; IL-1: Interleukin-1; NF-κB: nuclear factor kappa B; TGF-β: transforming-growth factor-beta; PGE2: prostaglandin E2; IDO: indoleamine 2, 3-dioxygenase; iNOS: inducible nitric-oxide synthase; TLRs: toll-like receptors; GvHD: graft-versus-host disease.

## Competing interests

The authors declare that they have no competing interests.

## Authors' contributions

ZP H, YY J, SS Z, Y L, LX W and YF S planned the manuscript outline. ZP H wrote the draft manuscript, YY J, SS Z and YL revised the manuscript, LX W and YF S finalized the manuscript. All authors read and approve the final manuscript.
